# 1-(Benzothia­zol-2-yl)-3-(4-nitro­benzo­yl)thio­urea

**DOI:** 10.1107/S1600536809030803

**Published:** 2009-08-08

**Authors:** Sohail Saeed, Naghmana Rashid, Rizwan Hussain, Peter G. Jones

**Affiliations:** a Department of Chemistry, Research Complex, Allama Iqbal Open University, Islamabad, Pakistan; b Directorate of Chemical & Power Sources, National Development Complex, PO Box 2216, Islamabad, Pakistan; c Institut für Anorganische und Analytische Chemie, Technische Universität Braunschweig, Postfach 3329, 38023 Braunschweig, Germany

## Abstract

The mol­ecule of the title compound, C_15_H_10_N_4_O_3_S_2_, is almost planar (r.m.s. deviation = 0.1Å for all non-H atoms). An intra­molecular N—H⋯O=C hydrogen bond is observed. In the crystal, mol­ecules are connected into layers parallel to (10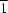) by a classical inter­molecular hydrogen bond from the second NH group to a nitro O atom and by three weak hydrogen bonds of the C—H⋯*X* type (*X* = O or S_thione_).

## Related literature

For general background to the chemistry of thio­urea derivatives, see Choi *et al.* (2008 ▶); Jones *et al.* (2008 ▶); Su *et al.* (2006 ▶). For related structures, see: Saeed *et al.* (2008*a* ▶,*b* ▶,*c* ▶); Yunus *et al.* (2008 ▶).
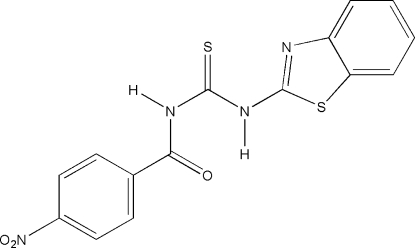


## Experimental

### 

#### Crystal data

C_15_H_10_N_4_O_3_S_2_
*M* *_r_* = 358.39
Monoclinic, 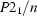

*a* = 7.1596 (3) Å
*b* = 17.9071 (5) Å
*c* = 11.5768 (4) Å
β = 96.446 (4)°
*V* = 1474.85 (9) Å^3^
*Z* = 4
Cu *K*α radiation
μ = 3.50 mm^−1^
*T* = 100 K
0.20 × 0.10 × 0.05 mm

#### Data collection

Oxford Diffraction Xcalibur Nova A diffractometer
Absorption correction: multi-scan (*CrysAlis Pro*; Oxford Diffraction, 2009 ▶) *T*_min_ = 0.682, *T*_max_ = 1.000 (expected range = 0.573–0.840)
30943 measured reflections
3026 independent reflections
2834 reflections with *I* > 2σ(*I*)
*R*_int_ = 0.040

#### Refinement

*R*[*F* ^2^ > 2σ(*F* ^2^)] = 0.029
*wR*(*F* ^2^) = 0.078
*S* = 1.06
3026 reflections
225 parameters
H atoms treated by a mixture of independent and constrained refinement
Δρ_max_ = 0.28 e Å^−3^
Δρ_min_ = −0.25 e Å^−3^

### 

Data collection: *CrysAlis Pro* (Oxford Diffraction, 2009 ▶); cell refinement: *CrysAlis Pro*; data reduction: *CrysAlis Pro*; program(s) used to solve structure: *SHELXS97* (Sheldrick, 2008 ▶); program(s) used to refine structure: *SHELXL97* (Sheldrick, 2008 ▶); molecular graphics: *XP* (Siemens, 1994 ▶); software used to prepare material for publication: *SHELXL97*.

## Supplementary Material

Crystal structure: contains datablocks I, global. DOI: 10.1107/S1600536809030803/im2131sup1.cif

Structure factors: contains datablocks I. DOI: 10.1107/S1600536809030803/im2131Isup2.hkl

Additional supplementary materials:  crystallographic information; 3D view; checkCIF report

## Figures and Tables

**Table 1:** Hydrogen-bond geometry (Å, °)

*D*—H⋯*A*	*D*—H	H⋯*A*	*D*⋯*A*	*D*—H⋯*A*
N1—H01⋯O1	0.83 (2)	1.92 (2)	2.598 (2)	138 (2)
N2—H02⋯O2^i^	0.84 (2)	2.42 (2)	3.261 (2)	175 (2)
C5—H5⋯O1^ii^	0.95	2.55	3.462 (2)	161
C11—H11⋯O2^i^	0.95	2.41	3.318 (2)	159
C12—H12⋯S2^iii^	0.95	2.73	3.673 (1)	173
C7—H7⋯S2^iv^	0.95	2.91	3.563 (1)	127

Symmetry codes: (i) 
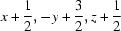
; (ii) 
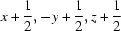
; (iii) 
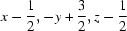
; (iv) 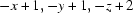.

**Fig. 1.**
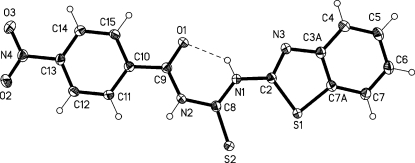
The molecular structure of the title compound in the crystal. Ellipsoids correspond to 50% probability levels.

**Fig. 2.**
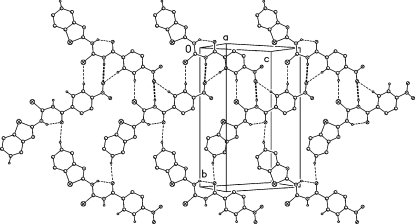
Packing diagram of the title compound viewed perpendicular to (101). Thin dashed lines represent "weak" and thick dashed lines classical H bonds. H atoms not involved in H bonds are omitted for clarity.

## supplementary crystallographic information

### Comment

Thiourea and its derivatives have found extensive applications in the field of medicine, agriculture and analytical chemistry. They are known to exhibit a wide variety of biological activities such as antiviral, anti-bacterial, antifungal, antitubercular, herbicidal, insecticidal and some epoxy resin curing agents containing amino functional groups (Saeed*et al.*, 2008a,b,c). They have found broad areas of application *e.g.* in anion recognition, nonlinear optics and catalysis, and also display good coordination abilities (Choi *et al.*, 2008; Jones *et al.*, 2008; Su *et al.*, 2006). As part of our research on coordination chemistry of thioureas, we are interested in the study of the influence of non-covalent interactions, especially hydrogen bonds and π-π stacking interactions, on the coordination modes of benzothiazoles bearing the 4- nitrobenzoylthiourea group with transition metal ions. Such coordination compounds of thiourea have been studied for various biological systems like antibactrial, antifungal and anticancer activities (Yunus *et al.*, 2008).The importance of such work lies in the possibility that the next generation of thiourea derivatives might be more efficacious as antimicrobial and anticancer agents. However, a thorough investigation relating structure and activity of thiourea derivatives as well as their stability under biological conditions is required. These detailed investigations could be helpful in designing more potent antimicrobial and anticancer agents for therapeutic use. Condensation of acyl or aroyl thiocyanates with primary amines affords 1, 3-disubstituted thioureas in excellent yields in a single step. In the present paper, the crystal structure of the title compound is reported.

The molecule of the title compound is shown in Fig. 1. The molecule is approximately planar (r.m.s. deviation for all non-H atoms 0.1 Å). The two ring systems (S1–C7A plus N1, C8, N2; C10–C15 plus N4, C9) are essentially parallel (interplanar angle 1.06 (3)°), because non-zero torsion angles such as C11—C10—C9—N2 - 10.7 (2) and N1—C8—N2—C9 7.7 (2)° effectively cancel out.

An intramolecular hydrogen bond N1—H01···O1 is observed. The second classical H bond N2—H02···O2 combines with the three shortest "weak" H bonds H5···O1, H11···O2 and H12···S2 (Table 1) to form layers parallel to (101) (Fig. 2).

### Experimental

A mixture of ammonium thiocyanate (0.1 mol) and 4-nitrobenzoyl chloride (0.1 mol) in anhydrous acetone (60 ml) was stirred for 40 min. 2-Aminobenzothiazole (0.1 mol) was added and the reaction mixture was refluxed for 2 h. After cooling, the reaction mixture was poured into 800 ml of acidified cold water (pH = 5). The resulting dark yellow solid was filtered and washed with cold acetone (yield 1.56 g, 87%). The title compound (I) was obtained as suitable crystals for X-ray analysis after recrystallization of the solid from a 1:1 ethanol- dichloromethane mixture.

### Refinement

NH H atoms were refined freely. Other H atoms were placed in calculated positions and refined using a riding model with C—H 0.95 Å; hydrogen *U* values were fixed at 1.2 × *U*(eq) of the parent atom. Data are 99.3% complete to 2θ 145°.

### Crystal data

**Table:**

C_15_H_10_N_4_O_3_S_2_	*F*(000) = 736
*M**_r_* = 358.39	*D*_x_ = 1.614 Mg m^−^^3^
Monoclinic, *P*2_1_/*n*	Cu *K*α radiation, λ = 1.54184 Å
Hall symbol: -P 2yn	Cell parameters from 21284 reflections
*a* = 7.1596 (3) Å	θ = 3.8–75.7°
*b* = 17.9071 (5) Å	µ = 3.50 mm^−^^1^
*c* = 11.5768 (4) Å	*T* = 100 K
β = 96.446 (4)°	Lath, yellow
*V* = 1474.85 (9) Å^3^	0.20 × 0.10 × 0.05 mm
*Z* = 4	

### Data collection

**Table:**

Oxford Diffraction Xcalibur Nova A diffractometer	3026 independent reflections
Radiation source: Nova (Cu) X-ray Source	2834 reflections with *I* > 2σ(*I*)
mirror	*R*_int_ = 0.040
Detector resolution: 10.3543 pixels mm^-1^	θ_max_ = 75.9°, θ_min_ = 4.6°
ω scans	*h* = −8→9
Absorption correction: multi-scan (*CrysAlis PRO*; Oxford Diffraction, 2009)	*k* = −22→22
*T*_min_ = 0.682, *T*_max_ = 1.000	*l* = −14→14
30943 measured reflections	

### Refinement

**Table:**

Refinement on *F*^2^	Primary atom site location: structure-invariant direct methods
Least-squares matrix: full	Secondary atom site location: difference Fourier map
*R*[*F*^2^ > 2σ(*F*^2^)] = 0.029	Hydrogen site location: inferred from neighbouring sites
*wR*(*F*^2^) = 0.078	H atoms treated by a mixture of independent and constrained refinement
*S* = 1.06	*w* = 1/[σ^2^(*F*_o_^2^) + (0.0427*P*)^2^ + 0.7178*P*] where *P* = (*F*_o_^2^ + 2*F*_c_^2^)/3
3026 reflections	(Δ/σ)_max_ = 0.001
225 parameters	Δρ_max_ = 0.28 e Å^−^^3^
0 restraints	Δρ_min_ = −0.24 e Å^−^^3^

### Special details

**Table:**

Geometry. All e.s.d.'s (except the e.s.d. in the dihedral angle between two l.s. planes) are estimated using the full covariance matrix. The cell e.s.d.'s are taken into account individually in the estimation of e.s.d.'s in distances, angles and torsion angles; correlations between e.s.d.'s in cell parameters are only used when they are defined by crystal symmetry. An approximate (isotropic) treatment of cell e.s.d.'s is used for estimating e.s.d.'s involving l.s. planes.
Refinement. Refinement of *F*^2^ against ALL reflections. The weighted *R*-factor *wR* and goodness of fit *S* are based on *F*^2^, conventional *R*-factors *R* are based on *F*, with *F* set to zero for negative *F*^2^. The threshold expression of *F*^2^ > σ(*F*^2^) is used only for calculating *R*-factors(gt) *etc*. and is not relevant to the choice of reflections for refinement. *R*-factors based on *F*^2^ are statistically about twice as large as those based on *F*, and *R*- factors based on ALL data will be even larger.

### Fractional atomic coordinates and isotropic or equivalent isotropic displacement parameters (Å^2^)

**Table:**

	*x*	*y*	*z*	*U*_iso_*/*U*_eq_	
S1	0.53606 (4)	0.470055 (17)	0.84776 (3)	0.01726 (10)	
C2	0.44766 (17)	0.43337 (7)	0.71218 (11)	0.0165 (3)	
N3	0.45826 (16)	0.36156 (6)	0.69808 (10)	0.0194 (2)	
C3A	0.54057 (19)	0.32891 (8)	0.80022 (12)	0.0190 (3)	
C4	0.5778 (2)	0.25256 (8)	0.81532 (13)	0.0232 (3)	
H4	0.5452	0.2182	0.7538	0.028*	
C5	0.6629 (2)	0.22814 (8)	0.92178 (13)	0.0248 (3)	
H5	0.6894	0.1765	0.9331	0.030*	
C6	0.7108 (2)	0.27842 (8)	1.01331 (12)	0.0234 (3)	
H6	0.7681	0.2602	1.0859	0.028*	
C7	0.67605 (19)	0.35414 (8)	0.99963 (12)	0.0209 (3)	
H7	0.7094	0.3883	1.0614	0.025*	
C7A	0.59024 (18)	0.37870 (7)	0.89188 (12)	0.0178 (3)	
S2	0.43025 (6)	0.610190 (19)	0.71210 (3)	0.02877 (12)	
N1	0.36489 (16)	0.47552 (6)	0.61966 (10)	0.0173 (2)	
H01	0.316 (3)	0.4498 (11)	0.5646 (16)	0.029 (5)*	
N2	0.25433 (16)	0.57570 (6)	0.50564 (10)	0.0179 (2)	
H02	0.260 (3)	0.6224 (11)	0.5014 (16)	0.028 (5)*	
N4	−0.17337 (16)	0.67589 (7)	0.00397 (10)	0.0215 (2)	
O1	0.15674 (15)	0.46515 (5)	0.42156 (8)	0.0227 (2)	
O2	−0.20909 (16)	0.74281 (6)	0.00649 (9)	0.0300 (3)	
O3	−0.19731 (17)	0.63730 (6)	−0.08377 (9)	0.0314 (3)	
C8	0.34765 (18)	0.55045 (7)	0.61124 (12)	0.0183 (3)	
C9	0.16239 (18)	0.53355 (7)	0.41710 (11)	0.0174 (3)	
C10	0.07004 (18)	0.57425 (7)	0.31352 (11)	0.0168 (3)	
C11	0.04521 (19)	0.65149 (7)	0.30881 (12)	0.0194 (3)	
H11	0.0855	0.6812	0.3749	0.023*	
C12	−0.03826 (19)	0.68511 (8)	0.20783 (12)	0.0200 (3)	
H12	−0.0567	0.7376	0.2040	0.024*	
C13	−0.09376 (18)	0.64017 (7)	0.11316 (11)	0.0177 (3)	
C14	−0.07437 (19)	0.56315 (8)	0.11565 (11)	0.0188 (3)	
H14	−0.1166	0.5338	0.0495	0.023*	
C15	0.00793 (19)	0.53013 (7)	0.21676 (12)	0.0186 (3)	
H15	0.0224	0.4774	0.2207	0.022*	

### Atomic displacement parameters (Å^2^)

**Table:**

	*U*^11^	*U*^22^	*U*^33^	*U*^12^	*U*^13^	*U*^23^
S1	0.01993 (17)	0.01434 (16)	0.01670 (17)	0.00078 (11)	−0.00157 (12)	0.00075 (10)
C2	0.0163 (6)	0.0159 (6)	0.0173 (6)	−0.0001 (5)	0.0017 (5)	0.0013 (5)
N3	0.0218 (6)	0.0165 (6)	0.0194 (5)	0.0011 (4)	−0.0001 (4)	0.0017 (4)
C3A	0.0190 (6)	0.0186 (7)	0.0192 (6)	0.0002 (5)	0.0017 (5)	0.0028 (5)
C4	0.0278 (7)	0.0171 (7)	0.0240 (7)	0.0003 (5)	0.0005 (6)	0.0008 (5)
C5	0.0277 (7)	0.0180 (7)	0.0284 (7)	0.0024 (5)	0.0022 (6)	0.0072 (6)
C6	0.0237 (7)	0.0235 (7)	0.0222 (7)	0.0015 (5)	−0.0001 (5)	0.0076 (5)
C7	0.0205 (7)	0.0224 (7)	0.0192 (6)	0.0001 (5)	−0.0002 (5)	0.0018 (5)
C7A	0.0161 (6)	0.0164 (6)	0.0209 (6)	0.0011 (5)	0.0021 (5)	0.0023 (5)
S2	0.0467 (2)	0.01438 (18)	0.02184 (19)	−0.00151 (14)	−0.01103 (15)	0.00050 (12)
N1	0.0206 (6)	0.0145 (5)	0.0159 (5)	0.0002 (4)	−0.0023 (4)	0.0008 (4)
N2	0.0224 (6)	0.0122 (5)	0.0183 (5)	0.0002 (4)	−0.0014 (4)	0.0016 (4)
N4	0.0201 (6)	0.0226 (6)	0.0206 (6)	−0.0019 (4)	−0.0025 (4)	0.0037 (5)
O1	0.0314 (5)	0.0138 (4)	0.0214 (5)	0.0004 (4)	−0.0037 (4)	0.0006 (4)
O2	0.0372 (6)	0.0211 (5)	0.0294 (6)	0.0048 (4)	−0.0073 (5)	0.0060 (4)
O3	0.0439 (7)	0.0301 (6)	0.0182 (5)	−0.0027 (5)	−0.0059 (4)	−0.0006 (4)
C8	0.0190 (6)	0.0174 (6)	0.0182 (6)	0.0002 (5)	0.0012 (5)	0.0023 (5)
C9	0.0177 (6)	0.0168 (6)	0.0179 (6)	0.0006 (5)	0.0027 (5)	0.0000 (5)
C10	0.0156 (6)	0.0162 (6)	0.0186 (6)	−0.0008 (5)	0.0019 (5)	0.0008 (5)
C11	0.0220 (7)	0.0160 (6)	0.0190 (6)	−0.0003 (5)	−0.0024 (5)	−0.0019 (5)
C12	0.0215 (6)	0.0149 (6)	0.0227 (7)	0.0008 (5)	−0.0015 (5)	0.0002 (5)
C13	0.0162 (6)	0.0194 (6)	0.0168 (6)	0.0001 (5)	−0.0007 (5)	0.0026 (5)
C14	0.0191 (6)	0.0194 (6)	0.0178 (6)	−0.0012 (5)	0.0011 (5)	−0.0018 (5)
C15	0.0204 (6)	0.0143 (6)	0.0210 (7)	−0.0006 (5)	0.0015 (5)	−0.0005 (5)

### Geometric parameters (Å, °)

**Table:**

S1—C7A	1.7443 (13)	O1—C9	1.2269 (16)
S1—C2	1.7529 (13)	C9—C10	1.4927 (18)
C2—N3	1.2994 (18)	C10—C11	1.3949 (19)
C2—N1	1.3877 (17)	C10—C15	1.4017 (19)
N3—C3A	1.3896 (17)	C11—C12	1.3890 (18)
C3A—C4	1.4002 (19)	C12—C13	1.3815 (19)
C3A—C7A	1.4009 (19)	C13—C14	1.3863 (19)
C4—C5	1.383 (2)	C14—C15	1.3828 (19)
C5—C6	1.403 (2)	C4—H4	0.9500
C6—C7	1.385 (2)	C5—H5	0.9500
C7—C7A	1.3982 (18)	C6—H6	0.9500
S2—C8	1.6439 (14)	C7—H7	0.9500
N1—C8	1.3499 (17)	N1—H01	0.832 (19)
N2—C9	1.3791 (17)	N2—H02	0.84 (2)
N2—C8	1.4007 (17)	C11—H11	0.9500
N4—O3	1.2246 (16)	C12—H12	0.9500
N4—O2	1.2264 (16)	C14—H14	0.9500
N4—C13	1.4731 (16)	C15—H15	0.9500
			
C7A—S1—C2	87.52 (6)	C15—C10—C9	116.03 (11)
N3—C2—N1	117.86 (12)	C12—C11—C10	120.24 (12)
N3—C2—S1	117.58 (10)	C13—C12—C11	118.26 (12)
N1—C2—S1	124.55 (10)	C12—C13—C14	122.94 (12)
C2—N3—C3A	109.55 (11)	C12—C13—N4	118.49 (12)
N3—C3A—C4	125.00 (13)	C14—C13—N4	118.56 (12)
N3—C3A—C7A	115.09 (12)	C15—C14—C13	118.37 (12)
C4—C3A—C7A	119.89 (12)	C14—C15—C10	120.15 (12)
C5—C4—C3A	118.59 (13)	C5—C4—H4	120.7
C4—C5—C6	121.06 (13)	C3A—C4—H4	120.7
C7—C6—C5	121.08 (13)	C4—C5—H5	119.5
C6—C7—C7A	117.75 (13)	C6—C5—H5	119.5
C7—C7A—C3A	121.62 (12)	C7—C6—H6	119.5
C7—C7A—S1	128.12 (11)	C5—C6—H6	119.5
C3A—C7A—S1	110.23 (10)	C6—C7—H7	121.1
C8—N1—C2	128.62 (12)	C7A—C7—H7	121.1
C9—N2—C8	127.82 (11)	C8—N1—H01	117.8 (13)
O3—N4—O2	124.13 (12)	C2—N1—H01	113.5 (13)
O3—N4—C13	118.13 (11)	C9—N2—H02	121.6 (13)
O2—N4—C13	117.74 (11)	C8—N2—H02	110.6 (13)
N1—C8—N2	114.50 (12)	C12—C11—H11	119.9
N1—C8—S2	124.95 (10)	C10—C11—H11	119.9
N2—C8—S2	120.54 (10)	C13—C12—H12	120.9
O1—C9—N2	122.03 (12)	C11—C12—H12	120.9
O1—C9—C10	120.50 (12)	C15—C14—H14	120.8
N2—C9—C10	117.47 (11)	C13—C14—H14	120.8
C11—C10—C15	120.00 (12)	C14—C15—H15	119.9
C11—C10—C9	123.96 (12)	C10—C15—H15	119.9
			
C7A—S1—C2—N3	−1.03 (11)	C9—N2—C8—N1	7.7 (2)
C7A—S1—C2—N1	177.99 (12)	C9—N2—C8—S2	−173.54 (11)
N1—C2—N3—C3A	−178.55 (11)	C8—N2—C9—O1	−1.8 (2)
S1—C2—N3—C3A	0.54 (15)	C8—N2—C9—C10	178.80 (12)
C2—N3—C3A—C4	−178.51 (13)	O1—C9—C10—C11	169.88 (13)
C2—N3—C3A—C7A	0.44 (16)	N2—C9—C10—C11	−10.69 (19)
N3—C3A—C4—C5	179.12 (13)	O1—C9—C10—C15	−10.64 (19)
C7A—C3A—C4—C5	0.2 (2)	N2—C9—C10—C15	168.79 (12)
C3A—C4—C5—C6	0.3 (2)	C15—C10—C11—C12	−1.0 (2)
C4—C5—C6—C7	−0.6 (2)	C9—C10—C11—C12	178.44 (12)
C5—C6—C7—C7A	0.5 (2)	C10—C11—C12—C13	−0.5 (2)
C6—C7—C7A—C3A	0.0 (2)	C11—C12—C13—C14	1.8 (2)
C6—C7—C7A—S1	−177.83 (11)	C11—C12—C13—N4	−176.88 (12)
N3—C3A—C7A—C7	−179.35 (12)	O3—N4—C13—C12	169.77 (12)
C4—C3A—C7A—C7	−0.3 (2)	O2—N4—C13—C12	−9.23 (19)
N3—C3A—C7A—S1	−1.19 (15)	O3—N4—C13—C14	−8.94 (19)
C4—C3A—C7A—S1	177.82 (11)	O2—N4—C13—C14	172.05 (12)
C2—S1—C7A—C7	179.17 (13)	C12—C13—C14—C15	−1.4 (2)
C2—S1—C7A—C3A	1.17 (10)	N4—C13—C14—C15	177.23 (12)
N3—C2—N1—C8	−175.86 (13)	C13—C14—C15—C10	−0.2 (2)
S1—C2—N1—C8	5.1 (2)	C11—C10—C15—C14	1.4 (2)
C2—N1—C8—N2	−179.42 (12)	C9—C10—C15—C14	−178.12 (12)
C2—N1—C8—S2	1.9 (2)		

### Hydrogen-bond geometry (Å, °)

**Table:**

*D*—H···*A*	*D*—H	H···*A*	*D*···*A*	*D*—H···*A*
N1—H01···O1	0.83 (2)	1.92 (2)	2.598 (2)	138 (2)
N2—H02···O2^i^	0.84 (2)	2.42 (2)	3.261 (2)	175 (2)
C5—H5···O1^ii^	0.95	2.55	3.462 (2)	161
C11—H11···O2^i^	0.95	2.41	3.318 (2)	159
C12—H12···S2^iii^	0.95	2.73	3.673 (1)	173
C7—H7···S2^iv^	0.95	2.91	3.563 (1)	127

Symmetry codes: (i) *x*+1/2, −*y*+3/2, *z*+1/2; (ii) *x*+1/2, −*y*+1/2, *z*+1/2; (iii) *x*−1/2, −*y*+3/2, *z*−1/2; (iv) −*x*+1, −*y*+1, −*z*+2.
